# High Ratio of Dietary Palmitic Acid to DHA + EPA Induces Glucose Metabolic Disorder Through Endocrine and Transcriptional Regulation in Large Yellow Croaker (*Larimichthys crocea*)

**DOI:** 10.3390/metabo16010072

**Published:** 2026-01-13

**Authors:** Qi Wang, Huaicheng Ge, Zhixiang Gu, Hao Chen, Hua Mu, Kangsen Mai, Wenbing Zhang

**Affiliations:** 1 Key Laboratory of Aquaculture Nutrition and Feed (Ministry of Agriculture and Rural Affairs) & Key Laboratory of Mariculture (Ministry of Education), Fisheries College, Ocean University of China, Qingdao 266003, China; 13094755457@163.com (Q.W.); 17669662398@163.com (H.G.); gzxianga@163.com (Z.G.); chenhao2412@stu.ouc.edu.cn (H.C.); huamu@jou.edu.cn (H.M.); kmai@ouc.edu.cn (K.M.); 2 Shenzhen Institute, Ocean University of China, Shenzhen 518100, China

**Keywords:** glyceryl palmitate, *Larimichthys crocea*, glycolipid metabolism, fatty acids, adipokines

## Abstract

**Background/Objectives**: Replacing fish oil with vegetable oil is an important measure for aquaculture to relieve the pressure of fish oil, but it is also easy to cause the growth decline and metabolic disorder of farmed animals, mainly due to the change in dietary fatty acids. This study investigated the regulatory effects of dietary fatty acid composition on glucose metabolism in large yellow croaker (*Larimichthys crocea*) with an initial weight of 30.51 ± 0.16 g. **Methods**: Three isonitrogenous (~43% crude protein) and isolipid (~11% crude lipid) diets were formulated as follows: control (CON, DHA/EPA-rich oil as primary lipid), moderate palmitic acid (MPA, 50% of DHA+EPA-rich oil was replaced by glyceryl palmitate), and high palmitic acid (HPA, 100% of DHA+EPA-rich oil was replaced by glyceryl palmitate). **Results**: After 10 weeks of feeding, the HPA significantly reduced the liver/muscle glycogen contents, increased the liver lipid content, decreased the serum leptin/insulin level, and increased the adiponectin level. The levels of DHA and EPA in liver were decreased significantly. Transcriptionally, HPA upregulated hepatic glucokinase (*gk*, glycolysis) but down-regulated glycogen synthase (*gys*) and *insulin*/*irs2* (insulin pathway) while inhibiting muscle *ampk* and leptin receptor (*lepr*). **Conclusions**: This study showed that high dietary PA/(DHA + EPA) impacted glycolipid homeostasis through endocrine and transcriptional regulation, leading to increased crude lipid and decreased glycogen levels, which provides a theoretical basis for scientific aquatic feed fatty acid formulation.

## 1. Introduction

The growing conflict between global supply and demand of fish oil has made the development of alternative lipid sources a research priority in aquaculture [1]. However, the substitution of fish oil indeed changes the fatty acid profile of feed, which may have profound implications for the metabolic health of farmed species [2]. Numerous studies indicate that the changes in dietary fatty acid composition can significantly influence systemic glucose metabolism [3,4]. In mammalian studies, high dietary saturated fatty acids have been proven to reduce insulin sensitivity in liver [5] and neuron [6]. For example, palmitic acid induces insulin resistance by disrupting hepatic lipid metabolism and insulin secretion, while simultaneously blocking the phosphorylation of key signaling proteins like Akt, the insulin receptor, and IRS1 in HepG2 cells [7]. Therefore, the influence of fatty acid profiles on glucose metabolism warrants close attention when formulating aquafeeds with alternative lipid sources.

The link between dietary fatty acids and glucose metabolism is largely mediated by adipokine regulation [8,9]. High intake of saturated fats raises circulating non-esterified fatty acids (NEFA), which in turn blocks glucose oxidation and drives insulin resistance. Palmitic acid, for instance, is known to trigger hepatic inflammation—a phenomenon verified in species like silver pomfret (*Pampus argenteus*) [10] and zebrafish (*Danio rerio*) [11]. Over time, persistent inflammation and high NEFA levels disrupt the insulin signaling cascade. This results in flawed glucose transport and phosphorylation [12], which are more severe in fish with poor glucose tolerance [13]. N-6 polyunsaturated fatty acids (PUFAs) tend to induce inflammation and blunt insulin sensitivity, although these effects are not universal and can be highly dependent on environmental conditions and species-specific factors [14]. N-3 PUFAs do the opposite, acting as anti-inflammatory agents that boost insulin function [15,16]. This protective role is clearly seen in juvenile Nile tilapia (*Oreochromis niloticus*) [17]. Furthermore, adipokines such as leptin and adiponectin play critical regulatory roles in this process. Leptin targets the hypothalamus to fine-tune the insulin-glucose axis [18], a mechanism highly conserved in teleosts. Specifically, insulin and glucagon trigger the upregulation of leptin A. In turn, Leptin exerts a glucose-dependent biphasic regulation on insulin release: it inhibits insulin a (*insa*) expression under basal glucose conditions but stimulates *insa* expression under hyperglycemic conditions [19]. Adiponectin also functions as a metabolic enhancer in skeletal muscle, improving both insulin signaling and glucose utilization [20]. In grass carp, both adiponectin A and B suppress basal glycemia and enhance hepatic glycogen storage [21]. These findings highlight how adipokines serve as a functional bridge connecting fatty acid intake to systemic glucose regulation.

As a cornerstone of the Chinese marine aquaculture industry, the large yellow croaker maintains the highest annual yield among all farmed marine fish [22]. Its widespread appeal stems from a combination of delicate flavor and visually attractive golden pigmentation. The metabolic ability to generate long-chain polyunsaturated fatty acids (LC-PUFA) from LA and ALA is restricted in large yellow croaker, a trait shared by other marine carnivorous fish [23]. Because its physiological health hinges so heavily on the fatty acid composition of its diet, it offers a unique opportunity to study lipid metabolism in depth. However, comprehensive research evaluating how dietary fatty acid composition affects glucolipid metabolism remains scarce, particularly from the perspective of endocrine regulation. To accentuate the contrast between saturated fatty acids (glyceryl palmitate) and n-3 highly unsaturated fatty acids (n-3 HUFAs; EPA/DHA), we formulated diets using glyceryl palmitate alongside oils enriched with EPA and DHA [4]. This research investigates the metabolic interplay between palmitic acid and DHA/EPA using gradient dietary ratios. The purpose of this study is to map their regulatory effects on glucose homeostasis, paving the way for more precise fatty acid formulations in aquaculture.

## 2. Materials and Methods

### 2.1. Ethical Statement

Every animal handling and care method was performed under the supervision of Ocean University of China’s Animal Care Committee (OUC-AE-2025-308).

### 2.2. Diet Preparation

Three experimental diets were formulated to be isonitrogenous and isolipidic, containing approximately 43% crude protein and 11% crude lipid, respectively. Fish meal and soybean meal were utilized as the primary dietary protein sources. The control diet contained DHA+EPA-enriched oil as the main lipid sources. In the experimental diets, 50% and 100% of the DHA+EPA-enriched oil were replaced by glycerin palmitate, named MPA group and HPA group, respectively. The experimental diet details are presented in Table 1. The feed preparation procedure was as follows [4]: feed ingredients were first ground to pass through a 60-mesh sieve. the dry powders were thoroughly mixed step-by-step following the recipe, ensuring consistency via a high-performance mixer (0.5 V Type, Shanghai Tianxiang Jiantai Pharmaceutical Machinery Co., Ltd., China). Simultaneously, the weighed soybean lecithin was fully incorporated into the required oil mixture until clear. The oil containing dissolved lecithin was evenly sprayed onto the mixed powder with manual rubbing to ensure homogeneity. An appropriate amount of water was added, and the mixture was pelleted using a granulator (EL-260, Weihai Friendship Machinery Factory, Shandong, China) to produce pellets with a diameter of 5 mm. The pellets were placed on trays and dried in a blast dryer at 55 °C for 12 h. Upon drying, the finished pellets were stored at −20 °C prior to use. The specific fatty acid profiles of these diets are presented in Table 2.

### 2.3. Experimental Animals and Feeding Management

Juvenile large yellow croaker (*Larimichthys crocea*) were procured from the Ningde Fufa Fry Hatchery (Ningde, China) and transported to the aquaculture facility of Ningde Fufa Aquatic Products Co., Ltd. (Ningde, China) (temperature: 20.5–25.4 °C, salinity: 23.72–28.72‰). Upon arrival, the fish were acclimatized for two weeks in floating net cages (4.0 m × 4.0 m × 4.0 m). At this stage, fish were fed a control diet to adapt to the artificial compound diet. Following acclimation, 450 healthy fish of uniform size (initial weight: 30.51 ± 0.16 g) were fasted for 24 h and randomly distributed into three treatment groups. Net cages (2 m^3^, 1.0 m × 1.0 m × 2.0 m) with 50 fish per cage were used to establish each group in triplicate. The aquaculture cycle was 10 weeks. The fish were fed twice daily (05:00 and 17:00) with full food. During the feeding trial, water temperature and salinity fluctuated within the ranges of 20.5–25.4 °C and 23.72–28.72‰, respectively. Dissolved oxygen remained over 6 mg/L. Fish behavior and feeding status were monitored and recorded daily.

### 2.4. Sample Collection

After the 10-week feeding regimen, all survivors were deprived of feed for 24 h to clear intestinal contents. From each net cage, nine fish were randomly sampled and anesthetized by immersion in MS-222 (Shanghai Reagent Factory, China). Caudal vein blood was collected with syringe immediately upon anesthesia and maintained at 4 °C. Serum was isolated by centrifuging at 836× *g* (4 °C, 10 min), then aliquoted and snap-frozen in liquid nitrogen. Samples were stored at −80 °C prior to the quantification of various metabolic markers, including glucose, insulin, triglycerides, NEFA, leptin, and adiponectin. Liver and muscle samples were similarly excised, sealed in RNase-free tubes (Biosharp, Hefei, China), and frozen at −80 °C.

### 2.5. Determination of Glycogen and Lipid Content in the Liver and Muscle

Hepatic and muscular glycogen content was determined by commercial kits (Nanjing Jiancheng Bioengineering Institute, Jiangsu, China). Fresh tissue samples were weighed (≤100 mg) after being cleansed with physiological saline and blotted dry with filter paper. Samples were mixed with three volumes of alkaline solution and hydrolyzed in a boiling water bath for 20 min to yield glycogen hydrolysate. After adding double-distilled water to form the detection solution, color-developing reagent was introduced and mixed thoroughly. The mixture was then boiled for 5 min, followed by cooling under running water, and the absorbance was determined at 620 nm with a microplate reader (SpectraMax i3x, Shanghai, China) with the blank tube zeroed. Glycogen concentration was calculated by comparison with a standard curve.

The quantification of total lipids in liver and muscle tissues was performed using a modified chloroform-methanol method [24]. Prior to analysis, tissues were lyophilized using a freeze-dryer (ALPHA 1–2, Christ, Germany) until constant weight was achieved, and then homogenized. Samples (~100 mg) were treated with 4 mL of chloroform/methanol (2:1, *v*/*v*) and agitated for 24 h. Subsequently, another 2 mL of the solvent was introduced, and the supernatant was harvested following centrifugation (3000× *g*, 15 min). To ensure maximum recovery, the solid residue underwent a second extraction cycle with 2 mL of solvent, and the resulting liquid was added to the first fraction. The pooled extract was treated with 1.2 mL of 1.6% CaCl_2_ and agitated intensely, after which it was kept undisturbed overnight to allow the phases to stratify. The upper phase was discarded using a pipette and the lower organic phase was evaporated under a stream of nitrogen. The residue was further dried at 75 °C and lipid content was calculated gravimetrically.

### 2.6. Determination of Fatty Acid Composition in Liver and Muscle

Fatty acid methyl esters (FAMEs) were prepared following the method from Mu et al. [25]. Approximately 100 mg of dry powder was treated with 3 mL of KOH-methanol and heated at 75 °C for 20 min. After the mixture cooled down, 3 mL of HCl-methanol was added, and the sample was heated again at 75 °C for another 20 min. Once at room temperature, the lipids were extracted into 1 mL of n-hexane by vortexing. The mixture was allowed to sit overnight to separate the layers. The top layer was then collected, spun to remove particles, and analyzed by gas chromatography (GC, Santa Clara, CA, USA) to measure the fatty acids.

### 2.7. Determination of Serum Glucose and Serum Hormones

Serum glucose levels were tested using the glucose oxidase technique using a microplate reader (Multiskan GO, Thermo Fisher Scientific, Waltham, MA, USA). Serum hormone concentrations (adiponectin, insulin and leptin) were quantified by direct enzyme-linked immunosorbent assays (ELISAs) using commercial kits purchased from Cusabio (Wuhan, Hubei, China) according to the methods published before with a microplate reader (Multiskan GO, Thermo Fisher Scientific) [4].

### 2.8. RNA Extraction, Reverse Transcription, and Quantitative Real-Time PCR

We purified total RNA from the liver and muscle of large yellow croaker using TRIzol reagent (Vazyme, Nanjing, Jiangsu, China). The concentration and purity of the extracts were checked on a NanoDrop 2000 (Thermo Fisher Scientific, Waltham, MA, USA). Subsequently, the PrimeScript RT Kit (Takara, Dalian, China, RR047Q) was employed to generate cDNA from the RNA via a two-step reaction. Quantitative analysis was executed using Vazyme’s SYBR qPCR Master Mix (Nanjing, Jiangsu, China) and specific primers (see Supplementary Table S1). β-actin functioned as the housekeeping gene, selected for its invariant expression under the experimental conditions. Final data were analyzed using the 2^(−ΔΔCT)^ algorithm following amplification efficiency verification [26].

### 2.9. Data Statistics and Analysis

All values are expressed as the mean ± standard error of the mean (S.E.M.). Statistical processing was performed using the SPSS 20.0 software package. Group means were compared using a one-way ANOVA combined with Tukey’s multiple range test. Statistical significance was declared when the *p*-value was lower than 0.05.

## 3. Results

### 3.1. Hepatic and Muscular Glycogen and Lipid Levels in Large Yellow Croaker

**Figure 1:**
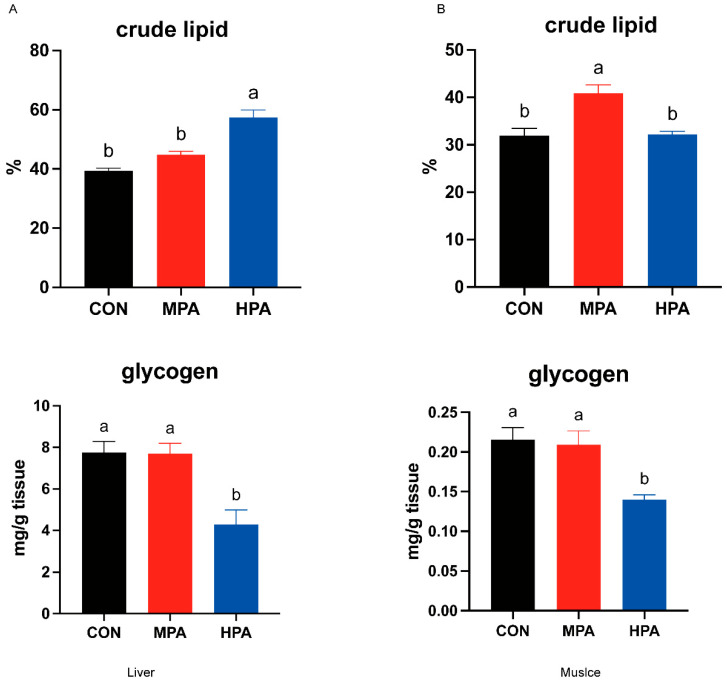
Effects of glycerin palmitate on hepatic and muscular glycogen and lipid levels in large yellow croaker (mean ± S.E.M., n = 3). (**A**) the contents of lipid and glycogen in the liver; (**B**) the contents of lipid and glycogen in the muscle. Following Tukey’s test, the same-letter bars show no discernible changes across treatments (*p* > 0.05).

After the experiment, the survival rate of large yellow croaker in all treatment groups was over 90% (91.33 ± 0.67 vs. 93.33 ± 2.40 vs. 90.00 ± 1.15; %), and there was no significant difference between the groups. Given the no significant effects on growth performance by glyceryl palmitate was found [27], the current study concentrated on the impacts on physiological and biochemical responses, especially on the glucose metabolism. As the proportion of glycerin palmitate in the diet increased, the hepatic glycogen content of large yellow croaker exhibited a decreasing trend. Compared to the CON group, the HPA group showed a significant reduction in hepatic glycogen content (*p* < 0.05, Figure 1A). Similarly, the HPA group’s muscle glycogen content was substantially lower than the CON group’s (*p* < 0.05, Figure 1B). Furthermore, the HPA group significantly increased hepatic lipid content (*p* < 0.05, Figure 1A). However, the MPA and CON groups did not significantly differ from one another (*p* > 0.05, Figure 1A). Regarding muscular lipid content, the MPA group had the highest level, which was significantly greater than those in the CON and HPA groups (*p* < 0.05, Figure 1B).

### 3.2. Serum Glucose, Lipid, and Hormone Levels in Large Yellow Croaker

**Figure 2:**
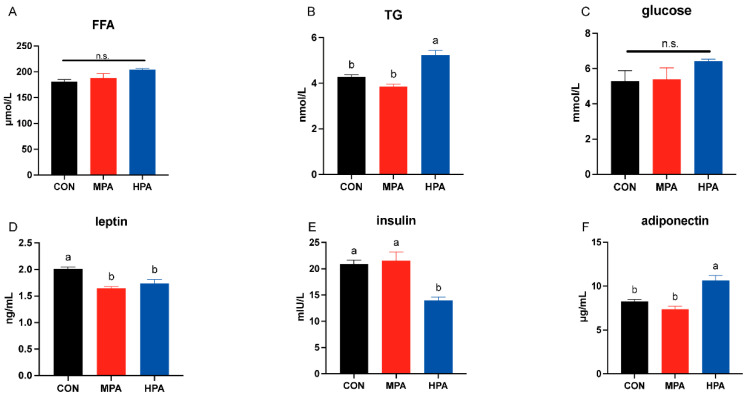
The serum glucose (**C**), lipid (**A**,**B**), and hormone (**D**–**F**) levels in large yellow croaker fed with different diets (mean ± S.E.M., n = 3). FFA: free fatty acid; TG: triglyceride; n.s.: no significance. Following Tukey’s test, the same-letter bars show no discernible changes across treatments (*p* > 0.05).

No significant changes were observed in serum glucose or free fatty acid (FFA) levels (*p* > 0.05, Figure 2A). However, serum triglyceride (TG) levels were found to be significantly higher in the HPA group compared to the CON group (*p* < 0.05, Figure 2B). Regarding adipokines, the administration of glyceryl palmitate resulted in a significant reduction in leptin (*p* < 0.05, Figure 2D), whereas adiponectin levels were significantly higher in the HPA group relative to the CON group (*p* < 0.05, Figure 2F). No significant difference in insulin was found between the MPA and CON groups (*p* > 0.05), but significantly lower levels were observed in the HPA group (*p* < 0.05, Figure 2E).

### 3.3. Hepatic Fatty Acid Composition

No significant changes were found in the levels of saturated fatty acids (SFA) or C20:1 in the liver (*p* > 0.05, Table 3). However, the composition of monounsaturated fatty acids (∑MUFA) was significantly altered. Specifically, a dose-dependent increase was seen in C16:1, C18:1n-9, and total ∑MUFA, and significantly higher levels were recorded in the HPA group compared to the CON and MPA groups. (*p* < 0.05). Conversely, C18:1n-7 followed an inverse trend, being significantly depleted in the HPA group relative to the CON and MPA treatments. Regarding polyunsaturated fatty acids, total n-3 PUFA levels were negatively correlated with dietary glyceryl palmitate; notably, the HPA group showed significantly reduced ∑n-3 PUFA and C22:6n-3 (DHA) levels compared to the other groups (*p* < 0.05, Table 3). Additionally, glyceryl palmitate significantly suppressed the content of C20:5n-3 (EPA). The PA/(DHA+EPA) ratio showed an increasing trend with higher dietary glyceryl palmitate, and was significantly elevated in the HPA group relative to the CON and MPA groups (*p* < 0.05, Table 3). Conversely, the total DHA+EPA content decreased with increasing glyceryl palmitate supplementation and was much lower in the HPA group.

### 3.4. Hepatic Gene Expression Profiles in Large Yellow Croaker

#### 3.4.1. Key Enzymes of Glucose Metabolism and Genes Related to Glucose Transport in Liver

**Figure 3:**
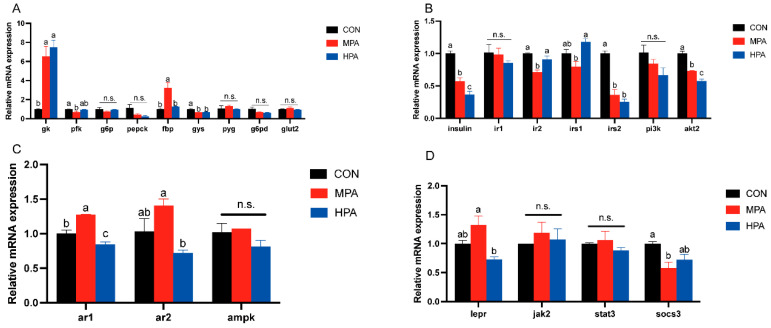
Hepatic gene expression profiles in large yellow croaker fed with different diets (mean ± S.E.M., n = 3). (**A**) Key enzymes of glucose metabolism and genes related to glucose transport. (**B**) Genes associated with insulin pathway. (**C**) Genes associated with adiponectin pathway. (**D**) Genes associated with leptin pathway. n.s.: no significance. Following Tukey’s test, the same-letter showed no discernible changes across treatments (*p* > 0.05), while different-letter showed significant changes across treatments (*p* < 0.05).

Figure 3A illustrates the hepatic transcriptional profiles related to glucose metabolism. Dietary glyceryl palmitate significantly modulated several key genes. Specifically, *gk* expression was upregulated in both MPA and HPA groups compared to the control (*p* < 0.05), showing a positive correlation with dietary inclusion. In contrast, *gys* mRNA levels were significantly suppressed in the treatment groups (*p* < 0.05). The MPA group specifically exhibited lower *pfk* expression but higher *fbp* levels relative to the CON group (*p* < 0.05). Although *pepck* appeared to decline with increasing supplementation, no statistically significant differences were found for *pepck* or *g6p* (*p* < 0.05). Similarly, the expression of *pyg*, *g6pd*, and *glut2* remained stable across all groups. However, *g6pd* displayed a decreasing trend in response to increasing dietary glyceryl palmitate.

#### 3.4.2. Insulin Pathway-Related Genes in Liver

Figure 3B depicted the hepatic mRNA profiles associated with the insulin signaling cascade. A dose-dependent downregulation was observed for *insulin*, *irs2*, and *akt2* in response to increasing dietary glyceryl palmitate. Notably, *irs2* transcription was significantly inhibited in the MPA group. Conversely, the expression levels of *ir1* and *pi3k* remained stable across all treatments.

#### 3.4.3. Adiponectin and Leptin Pathway-Related Genes in Liver

The expression patterns of genes associated with adiponectin and leptin pathway in the liver are displayed in Figure 3C,D. As the proportion of glyceryl palmitate increased, the expression of *ar1*, *ar2*, and *lepr* exhibited an initial increase followed by a decrease. MPA considerably increased *ar1* mRNA expression in comparison to the control group, whereas HPA significantly suppressed it. Even though there were no appreciable variations in the *ar2* and *lepr* mRNA expression between the glyceryl palmitate groups and the control, HPA significantly inhibited the expression of both *ar2* and *lepr* compared to the MPA group. The MPA group significantly inhibited the *socs3* mRNA level. In addition, glyceryl palmitate had no significant influence on the mRNA expression levels of *jak2* or *stat3*.

### 3.5. Muscular Gene Expression Profiles in Large Yellow Croaker

#### 3.5.1. Key Enzymes of Glucose Metabolism and Genes Related to Glucose Transport

**Figure 4:**
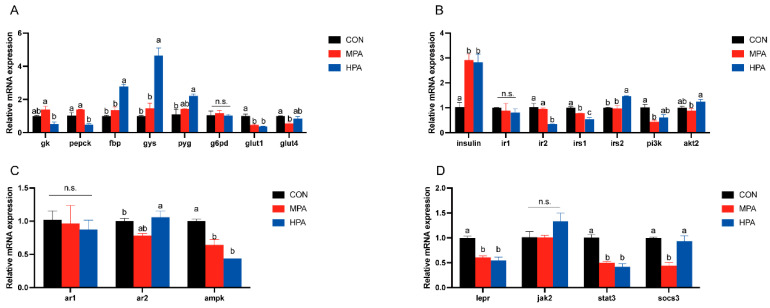
Muscular gene expression profiles in large yellow croaker fed with different diets (mean ± S.E.M., n = 3). (**A**) Key enzymes of glucose metabolism and genes related to glucose transport. (**B**) Genes associated with insulin pathway. (**C**) Genes associated with adiponectin pathway. (**D**) Genes associated with leptin pathway. n.s.: no significance. Following Tukey’s test, the same-letter showed no discernible changes across treatments (*p* > 0.05), while different-letter showed significant changes across treatments (*p* < 0.05).

Figure 4A illustrates the transcriptional profiles of glucose metabolism and transport genes in muscle tissue. A dose-dependent upregulation was observed for *fbp*, *gys*, and *pyg*, with the HPA group exhibiting significantly higher expression levels compared to the control (*p* < 0.05). Conversely, dietary glyceryl palmitate significantly suppressed *glut1* expression. Specific group effects were also noted: the HPA group showed a marked reduction in *pepck*, whereas the MPA group displayed significantly lower *glut4* levels. Meanwhile, *g6pd* expression remained stable across all treatments.

#### 3.5.2. Insulin Pathway-Related Genes

Figure 4B illustrates the transcriptional profiles of insulin pathway in muscle tissue. With increasing substitution levels of glyceryl palmitate, the mRNA expression levels of *insulin* and *irs2* exhibited a gradually increasing trend. Specifically, both MPA and HPA significantly up-regulated the expression of *insulin*, whereas only HPA significantly enhanced the expression of *irs2*. HPA significantly inhibited the mRNA expression of *ir2*, and glyceryl palmitate overall significantly suppressed the mRNA expression of *irs1*. In contrast, the expression of *pi3k* and *akt2* showed an initial decrease followed by an increase in response to rising substitution levels of glyceryl palmitate. However, *ir1* expression remained stable across all treatments.

#### 3.5.3. Adiponectin and Leptin Pathway-Related Genes

The expression patterns of genes related to adiponectin and leptin pathway in muscle are shown in Figure 4C,D. With increasing dietary inclusion levels of glyceryl palmitate, the mRNA expression levels of *ar2* and *socs3* exhibited a biphasic pattern characterized by an initial decrease followed by an increase. Among these, the *ar2* mRNA was significantly increased in the HPA group. In contrast, glyceryl palmitate did not exert significant effects on the mRNA expression of *ar1* or *jak2*. Additionally, glyceryl palmitate significantly suppressed the mRNA expression levels of *ampk*, *lepr*, and *stat3*.

## 4. Discussion

Due to the limited supply of fish oil resources, the substitution of fish oil with vegetable oils has become an inevitable trend [28,29]. Under these conditions, a risk of nutritional stress is posed to aquatic animals by changes in fatty acid profiles, and the negative effects of saturated fatty acids are particularly noted [30]. The proportion of fatty acids in feed did not affect the survival and growth of fish [31], this was also confirmed in this study. A complex connection between lipid and glucose metabolism is observed in living organisms [32,33]. It has been indicated that glucose balance in farmed animals can be disrupted by changes in dietary fats [34,35]. Since a limited ability to process carbohydrates is naturally possessed by fish [36], it is not yet clear if the reduced growth seen after switching to vegetable oils is linked to glucose metabolism issues. Therefore, glyceryl palmitate was selected as a representative saturated fat in this study, and the effect of the dietary saturated/polyunsaturated fatty acid ratio on glucose metabolism in large yellow croaker (*Larimichthys crocea*) was investigated. Hepatic and muscle glycogen levels were significantly reduced when DHA+EPA-rich oil was fully replaced by glyceryl palmitate. This finding is supported by observations in large yellow croaker, where muscle glycogen was decreased by the substitution of soybean oil [37]. The reduction in glycogen levels likely results from a dual mechanism of impaired synthesis and enhanced mobilization. First, the replacement of n-3 HUFAs depressed the PI3K/AKT pathway and downregulates glycogenesis [37]. Second, metabolic stress triggers glycogen depletion. As shown in carnivorous fish, hepatic glycogen is preferentially mobilized as the primary energy substrate when lipid utilization is suboptimal [38]. Additionally, dietary glyceryl palmitate significantly increased the crude lipid content in both muscle and liver tissues. Similar results have been reported in tilapia (*Oreochromis niloticus*) [39], grass carp (*Ctenopharyngodon idella*) [40], and blunt snout bream (*Megalobrama amblycephala*) [41]. The specific mechanism may be related to the overactivation of de novo fat synthesis by palmitic acid. The liver is recognized as the central organ for processing fats, and its fatty acid profile is known to be easily changed by the diet [42,43]. In this study, the total amount of saturated fatty acids (SFA) in the liver was not significantly altered by the addition of glyceryl palmitate, but the level of monounsaturated fatty acids (MUFA) was markedly increased. Since the harmful effects of high saturated fat levels are known to be reduced by MUFA [44,45], the observed rise in MUFA might be interpreted as an internal defense response triggered to keep liver cells healthy and metabolism balanced. Also, a drop in these polyunsaturated fats in the liver was directly caused by the lower amount of EPA and DHA provided in the feed. Because DHA and EPA cannot be easily produced by the large yellow croaker [46], it is held that adequate amounts must be supplied in the diet to maintain both muscle quality and overall health.

The regulation of glucose and fat metabolism is heavily relied upon by adipokines, which are proteins released by fat tissue [47]. Key roles in metabolism, inflammation, insulin response, and appetite control are played by these proteins [48]. In this experiment, serum leptin levels were significantly lowered by glyceryl palmitate. Given that leptin has been shown to promote fatty acid oxidation in aquatic animals [49], the observed lipid increase in these tissues may be attributed to an attenuated capacity for leptin-mediated lipid catabolism [50]. Serum adiponectin levels were significantly raised by glyceryl palmitate. This finding is supported by mammalian studies, where it has been noted that circulating adiponectin is markedly increased by diets high in saturated fats [51], which reflected the conservation of adiponectin regulation between fish and mammals. An essential role in the regulation of fat and sugar metabolism is played by adiponectin, a cytokine secreted by adipose tissue. It has been shown that lipid oxidation is promoted and fat formation is blocked by this protein [52]. Thus, the rise in adiponectin triggered by glyceryl palmitate might be viewed as a feedback response to abnormal fat storage. Insulin is regarded as a key hormone for blood sugar control [53]. In this study, serum insulin levels were reduced by glyceryl palmitate, and the ability of the large yellow croaker to regulate blood glucose was consequently impaired. Ultimately, it is suggested that the disturbance of glucose and lipid metabolism caused by high saturated fatty acids is partially explained by these hormonal changes.

Focusing on the signaling pathways of insulin, leptin, and adiponectin, this work uncovered how large yellow croaker responds to glyceryl palmitate at the transcriptional level. The findings provide insight into the underlying network linking hormonal signals to glucose homeostasis. In hepatic tissue, the expression of glucokinase (GK), the initial rate-limiting enzyme of the glycolytic pathway [54], was significantly activated by glyceryl palmitate treatment. At the same time, the transcriptional level of glycogen synthase (GYS), the key rate-limiting enzyme for glycogen synthesis, was significantly reduced [55]. The decrease in liver glycogen observed in this study was matched by the gene expression pattern. It is indicated that glycolysis is promoted and glycogen storage is inhibited by glyceryl palmitate, so the glucose balance in the liver is affected. Regarding the insulin signaling pathway, a significant drop in the expression of the *insulin* gene and its downstream molecule, *insulin receptor substrate 2* (IRS2), was caused by the treatment. This result is supported by reports in aquatic animals where insulin resistance is induced by saturated fatty acids [56]. It is suggested that insulin signal transmission might be disrupted by the activation of ceramide synthesis. The role of protein kinase B subtype 2 (AKT2) in improving insulin sensitivity is becoming better understood in fish. It is known that metabolic problems are treated by drugs like fenofibrate via AKT2 [57], so the value of this pathway is highlighted. In muscle tissue, insulin gene expression was raised by glyceryl palmitate. This change could be seen as a defense attempt to keep blood sugar balanced. However, the expression of the insulin receptor (IR2) and IRS1 was suppressed. It is indicated that the signaling path was blocked even with more insulin made, and the effective work of insulin was prevented. In the present study, we observed that glyceryl palmitate exerted a tissue-specific regulatory effect on the expression of genes related to the adiponectin and leptin signaling pathways. Specifically, it had no effect on *ampk* expression in the liver, whereas it significantly downregulated *ampk* expression in the muscle. This differential regulation is likely attributed to the distinct metabolic buffering capacities of the liver and muscle against palmitic acid. As the primary site for lipid oxidation, skeletal muscle has a limited capacity for lipid efflux, which renders it more susceptible to the conversion of excess palmitic acid into cytotoxic C16:0-ceramide under conditions of lipid overload [58]. In contrast, the liver can effectively clear excess lipids through its robust very-low-density lipoprotein (VLDL) secretion machinery, thereby alleviating the intracellular accumulation of lipotoxic substances. A similar tissue-specific regulatory pattern was also observed in the modulation of *stat3* by glyceryl palmitate. The decreased expression of the leptin receptor (LEPR) corresponded with reduced circulating leptin levels. Since fatty acid oxidation is promoted by leptin, increased fat storage might be explained by a weakening of the leptin signaling pathway. Overall, metabolic balance in the liver and muscle of large yellow croaker is affected by glyceryl palmitate. This is driven by the fact that the expression of key glucose metabolism enzymes, insulin signaling components, and energy sensors (AMPK and the leptin system) is regulated by the diet. Consequently, lipid deposition and growth performance are potentially modulated by these factors.

## 5. Conclusions

This study confirmed that a high dietary ratio of glyceryl palmitate (PA) to DHA+EPA disrupted glucolipid homeostasis in large yellow croaker. The imbalance induced by replacing fish oil with glyceryl palmitate did not merely alter tissue fatty acid profiles; it actively triggered metabolic disorders characterized by hepatic lipid accumulation and glycogen depletion. Mechanistically, we identified that this disruption was mediated through an endocrine axis involving suppressed insulin and leptin signaling, alongside elevated adiponectin, which subsequently upregulated glycolysis while inhibiting glycogen synthesis and insulin sensitivity at the transcriptional level. From an aquaculture industry perspective, these findings highlight a critical physiological limit in replacing fish oil with saturated fatty acid sources. To prevent metabolic disorders and fatty liver conditions, feed formulations must prioritize an optimal PA/(DHA+EPA) ratio rather than focusing solely on lipid energy provision. This study provides a theoretical basis for precision nutrition strategies to maintain the metabolic health and flesh quality of farmed marine fish.

## Figures and Tables

**Table 1:** Formulation and chemical proximate composition of the experimental diets (% dry matter).

Ingredients	CON ^a^	MPA ^b^	HPA ^c^
Fish meal ^d^	39.00	39.00	39.00
Soybean meal ^d^	21.70	21.70	21.70
Wheat meal ^d^	18.50	18.50	18.50
Beer yeast ^d^	1.50	1.50	1.50
Lecithin ^d^	2.00	2.00	2.00
Glycerin palmitate ^e^	0.00	2.50	5.00
DHA enriched oil + EPA enriched oil ^e^	5.00	2.50	0.00
Microcrystalline cellulose ^e^	10.35	10.35	10.35
Vitamin premix ^f^	1.00	1.00	1.00
Mineral premix ^g^	0.50	0.50	0.50
Attractant ^h^	0.30	0.30	0.30
Mold inhibitor ^i^	0.10	0.10	0.10
Ethoxyquin	0.05	0.05	0.05
Proximate analysis			
Crude protein	43.41	43.41	42.63
Crude lipid	10.69	10.63	10.67

^a^ CON: DHA+EPA-enriched oil group. ^b^ MPA: Glycerin palmitate replacing 50% of DHA+EPA-enriched oil. ^c^ HPA: Glycerin palmitate group. ^d^ Ingredients were purchased by Great Seven Biotechnology Co., Ltd., (Qingdao, China). The crude lipid of fish meal is 6.60% and the crude protein of fish meal is 70.27%; The crude lipid of soybean meal is 3.68% and the crude protein of soybean meal is 53.37%; The crude lipid of wheat flour is 1.58% and the crude protein of wheat flour is 19.69%. ^e^ Oil sources and microcrystalline cellulose in the study were purchased by Qingdao FuLin Biochemistry Co. Ltd, China. Glycerin palmitate, triglyceride type, purity > 85%; DHA enriched oil, triglyceride type, DHA: 70%, EPA: 10%; EPA enriched oil, triglyceride type, DHA: 22%, EPA: 33%. ^f^ Vitamin premix (mg/kg diet): ascorbic acid, 2000; α -tocopherol, 240; biotin, 60; cholecalciferol, 5; folic acid, 20; pantothenic acid, 60; inositol, 800; retinol acetate, 32; niacin acid, 200; riboflavin, 45; pyridoxine HCl,20; thiamin, 25; vitamin B12, 10; vitamin K3, 10; wheat middling, 16,473. ^g^ Mineral premix (mg/kg diet): Ca (H_2_PO_4_)_2_·H_2_O, 10,000; Ca (IO_3_)_2_·6H_2_O (1%), 60; ZnSO_4_·H_2_O, 50; CoCl_2_·6H_2_O (1%), 50; MnSO_4_·H_2_O, 45; CuSO_4_·5H_2_O, 10; FeSO_4_·H_2_O, 80; MgSO_4_·7H_2_O, 1200; Na_2_SeO_3_ (1%), 20; microcrystalline cellulose, 8485. ^h^ Attractant: In the present study, we used a mixture of glycine and betaine (*w*:*w* = 1:2) as attractant. ^i^ Mold inhibitor: In the present study, we used a mixture of calcium propionic acid and fumaric acid (*w*:*w* = 1:1) as mold inhibitor.

**Table 2:** Fatty acid composition of the experimental diets (% identified fatty acids).

Fatty Acid	CON ^a^	MPA ^b^	HPA ^c^
C14:0	1.03	1.35	1.24
C16:0	10.6	23.7	48.3
C18:0	3.51	6.05	6.52
C20:0	0.46	0.44	0.42
∑SFA ^d^	15.6	31.5	56.5
C16:1	1.48	1.95	1.40
C18:1n-7	2.64	2.31	1.64
C18:1n-9	8.55	10.8	13.7
C20:1	2.10	2.27	1.69
∑MUFA ^e^	14.8	17.3	18.4
C18:2n-6	15.5	16.7	14.7
C20:4n-6	1.03	0.73	0.30
∑n-6 PUFA ^f^	16.5	17.4	15.0
C18:3n-3	2.30	2.36	1.91
C20:5n-3	17.2	10.9	3.35
C22:6n-3	33.63	20.42	4.81
∑n-3 PUFA ^g^	53.1	33.7	10.1
DHA/EPA	1.96	1.87	1.43
PA ^h^/(DHA+EPA)	0.21	0.76	5.92
DHA+EPA	50.8	31.3	8.16

^a^ CON: DHA + EPA- enriched oil group. ^b^ MPA: Glycerin palmitate replacing 50% of DHA + EPA- enriched oil. ^c^ HPA: Glycerin palmitate group. ^d^ SFA: an abbreviation of saturated fatty acid. ^e^ MUFA: an abbreviation of monounsaturated fatty acid. ^f^ n-6 PUFA: an abbreviation of n-6 poly-unsaturated fatty acid. ^g^ n-3 PUFA: an abbreviation of n-3 poly-unsaturated fatty acid. ^h^ PA: an abbreviation of palmitic acid.

**Table 3:** Fatty acid composition in liver (% identified fatty acids).

	CON	MPA	HPA
C12:0	0.05 ± 0.01	0.05 ± 0.01	0.05 ± 0.00
C14:0	1.74 ± 0.22	1.38 ± 0.03	1.96 ± 0.11
C16:0	28.58 ± 0.60	30.91 ± 1.17	29.27 ± 1.56
C18:0	7.52 ± 0.27	8.31 ± 0.65	6.88 ± 0.05
C20:0	0.28 ± 0.01	0.38 ± 0.04	0.21 ± 0.06
ΣSFA	38.18 ± 0.92	41.02 ± 1.82	38.37 ± 0.55
C16:1	8.10 ± 0.43 ^b^	8.47 ± 0.15 ^b^	10.85 ± 0.82 ^a^
C18:1n-7	2.68 ± 0.10 ^a^	2.30 ± 0.10 ^a^	1.56 ± 0.20 ^b^
C18:1n-9	22.01 ± 1.33 ^b^	25.03 ± 0.53 ^b^	34.92 ± 0.97 ^a^
C20:1	0.98 ± 0.08	0.78 ± 0.06	1.08 ± 0.27
ΣMUFA	33.77 ± 1.64 ^b^	36.58 ± 0.50 ^b^	48.41 ± 1.32 ^a^
C18:2n-6	9.01 ± 1.27	7.52 ± 0.33	8.00 ± 0.12
C20:4n-6	0.77 ± 0.03 ^a^	1.02 ± 0.11 ^a^	0.35 ± 0.08 ^b^
Σn-6 PUFA	9.78 ± 1.27	8.54 ± 0.38	8.35 ± 0.19
C18:3n-3	1.05 ± 0.14	0.90 ± 0.05	1.11 ± 0.25
C20:5n-3	4.98 ± 0.09 ^a^	3.32 ± 0.41 ^b^	1.28 ± 0.12 ^c^
C22:6n-3	12.23 ± 0.38 ^a^	9.64 ± 1.44 ^a^	2.48 ± 0.24 ^b^
Σn-3 PUFA	18.26 ± 0.37 ^a^	13.86 ± 1.90 ^a^	4.87 ± 0.60 ^b^
DHA/EPA	2.45 ± 0.03 ^a^	2.89 ± 0.08 ^b^	1.94 ± 0.03 ^c^
PA /(DHA+EPA)	1.66 ± 0.03 ^b^	2.52 ± 0.48 ^b^	7.90 ± 0.60 ^a^
DHA+EPA	17.21 ± 0.47 ^a^	12.96 ± 1.86 ^a^	3.76 ± 0.36 ^b^

Following Tukey’s test, the same-letter showed no discernible changes across treatments (*p* > 0.05), while different-letter showed significant changes across treatments (*p* < 0.05).

## Data Availability

The original contributions presented in this study are included in the article/Supplementary Material. Further inquiries can be directed to the corresponding author.

## Supplementary Materials

The following supporting information can be downloaded at: https://www.mdpi.com/article/10.3390/metabo16010072/s1, Table S1: Primer table for Q-RT-PCR.
