# Supplementary material for: High Ratio of Dietary Palmitic Acid to DHA + EPA Induces Glucose Metabolic Disorder Through Endocrine and Transcriptional Regulation in Large Yellow Croaker (Larimichthys crocea)
Source: Metabolites. 2026 Jan 13;16(1):72. doi: 10.3390/metabo16010072 (PMC12844451; doi:10.3390/metabo16010072)
Supplement: Supplementary file 1 [file metabolites-16-00072-s001.zip › metabolites-4057725-supplementary.pdf]

Supplementary Table S1 Primer table for Q-RT-PCR

Target genes	Forward (5'-3')	Reverse (5' -3')
gk	GTGGGTGAAGATGACGAGAG	CGTACAGGAAAGGAAAAGGT
pfk	CCTGCTACCATCAGCAACA	TCTCCACCACAAACACTCG
pepck	GCCAGTATGAGGGTGATGA	GGTTACAGGGCCAGTTGTT
fbp	TCCTGTGTGCTGGTGTCTG	GGGCTCATCATCTGTGTTC
g6p	AACCAAGAAACCCACCAAC	CTTCAGCCACAATCATACC
g6pd	CCGTTTGGTACTCAGGGAC	CATCTGGAGCAAGTGGTTCT
gys	TGTGACGGTGGTTGTTTTC	CTCGGTCCAGGATGTTGTT
pyg	TGATCTGGAGCCGGAGAAG	TCGGTGACGAGGTCATTGA
glut1	ACCGCCATTCCCATCAAACC	CCCAGCCTGTCTATGCAACC
glut2	GTCGGTCTGGGAGGAATGT	CTATGAACCACGGAATGGG
glut4	ATTCCTCTACATCGTCCGC	CCATCTTCCTCTTCTCCTC
insulin	GTCTGCTCTGTCTCCACTTC	TCTCATCTCCACCTTCTGCT
ir1	CCCTATTTCCTCGTCCAAC	GCAACTTCATCCCTTTCTG
ir2	ACCCGTCTTCATACTCACA	CGTCCATTTTTCTTGCCAC
irs1	TGCGGGGGATTATGGAGTG	AGCAGCAGCGTCAGAGTTG
irs2	ACTGTCTCGTTCCCCACCC	AGAGCCAAACTCCTCGCAT
pi3k	CAAGGCTTCTGTCTGCTGG	GTGCGATATAACGTGGGGT
akt2	AGACCCCAAACAAAGGCTC	TCGTCATCGAAGTAACGCG
lepr	CATCCTCCTACAGCTTTGAGTG	GGATTTCTATCCAGCGTCATG
jak2	CCCTCACCAAACAGGACAAT	CCGGAGCCACCTCTTTACAC
stat3	TCACTAACCACCCAAAGAATG	GCTTAGGACCTCTGCCACTT
socs3	CAAGAAGAGGCACTGGATTAGG	CACATTGGTTAGCAGCAGAGG
ar1	AACATCCCGAACACTACCTT	ACTCACCTGTTAGGGCTCAT
ar2	CCTCAGCCCTGCTTCATCTA	TCAGACCCAGACCCACAAAC
ampk	ACAAGGCATACAGAATGACCC	TGCGAACAAGCTGTGGCTCT
β-actin	CTACGAGGGTTATGCCCTGCC	TGAAGGAGTAACCGCGCTCTGT

gk: glucokinase; *pfk*: fructose phosphate kinase liver type; *pepck*: phosphoenolpyruvate carboxykinase; *fbp*: fructose-1, 6-diphosphatase; *g6p*: glucose-6-phosphatase; *g6pd*: glucose-6-phosphate dehydrogenase; *gys*: glycogen synthase; *pyg*: glycogen phosphorylase; *glut1*: glucose transporter 1; *glut2*: the glucose transporter 2; *glut4*: the glucose transporter 4; *ir1*: insulin receptor 1; *ir2*: insulin receptor 2; *irs1*: insulin receptor substrate 1; *irs2*: insulin receptor substrate 2; *pi3k*: phosphatidylinositol 3-hydroxykinase; *akt2*: protein kinase B; *lepr*: leptin receptor; *jak2*: tyrosine kinase 2; *stat3*: cell signal transduction and transcriptional activator 3; *socs3*: cytokine signal transduction inhibitory molecule 3; *ar1*: adiponectin receptor 1; *ar2*: adiponectin receptor 2; *ampk*: adenyl-activated protein kinase.
